# Editorial: Robotic in-space servicing, assembly and manufacturing

**DOI:** 10.3389/frobt.2024.1421697

**Published:** 2024-08-09

**Authors:** David Barnhart, Rudranarayan Mukherjee, Mini Chakravarthini Rai, Nicholas D'Amore, Carl Glen Henshaw

**Affiliations:** ^1^ Department of Astronautical Engineering, University of Southern California, Los Angeles, CA, United States; ^2^ Jet Propulsion Laboratory, California Institute of Technology, Pasadena, CA, United States; ^3^ School of Engineering, University of Lincoln, Lincoln, England, United Kingdom; ^4^ Naval Research Laboratory, Washington, DC, United States

**Keywords:** robotics, space robotics and automation, compliance, assembly, large structure assembly

In-space assembly and manufacturing (or ISAM) represent the next frontier to building and maintaining space infrastructure. By assembling and manufacturing components directly in space, we may be able to overcome the limitations of launch vehicle size and payload capacity, enabling the creation of larger, more complex structures. This capability is crucial for advancing space exploration and utilization, as it allows for the construction of habitats, satellites, and other systems that are optimized for the space environment rather than being constrained by the need to survive the rigors of launch. In-space manufacturing also opens the door to utilizing local resources, such as lunar or asteroid materials, which can reduce the cost and increase the sustainability of long-term space missions.

The potential applications of in-space assembly and manufacturing are vast and varied. For instance, it can facilitate the development of large-scale solar power stations that can provide clean energy to Earth, the creation of advanced scientific instruments and telescopes that offer unprecedented views of the universe, and the construction of space habitats that support human life for extended periods. Moreover, this technology can enhance the repair and maintenance of existing satellites and space stations, extending their operational lifetimes and improving their performance.

Instantiation of almost any ISAM capability require robotics that are sophisticated and of enough fidelity and strength to translate to the large and varied manipulation applications envisioned. While “robots” have existed for some time on the International Space Station (ISS) through robotic arms and augmentation elements, the creation of autonomous independent robotic elements that can exist on free flying platforms is relatively new. The ability to manipulate in space translates to extending a single monolithic platforms primary function (i.e., a satellite) into a multi-purpose servicing platform that opens up incredible possibilities for expanding what is done in orbit, after launch.

The field of space robotics is an order of magnitude more complicated given the sheer difficulty of real time communications, visualization of objects, and compliance required to account for multiple degrees of freedom required to operate in the space environment. The articles included here are meant to offer some insight and thought on the various challenges and potential solutions to enabling various levels of dexterity, manipulation, and thus potential functions that on orbit space robotics can affect.

Relative to the challenges identified above there is a large trade space between “compliance” and “control” that enables both assembly of structures too large to fit into launch vehicle fairings today, as well as control down to micro-scale similar to the dexterity of a human hand. A mini article “Compliant Robotic Behaviours for Satellite Servicing” from Cressman et al. explores this specific problem where the space environment needs robots to cope with uncertainties, dynamics, and communication delays or interruptions, similar to human astronauts. They introduce a unique approach for compliant behaviors that could be applied to multiple types of robotic systems to address stiffness and dampening that drive a controller that introduces compliance.

Architecture changes to support very large structures are explored in “Built On-Orbit Robotically-Assembled Gigatruss (BORG): Mixed Assembly Architecture Trade Study” by Chapin et al. The problem of launch vehicle volumetric limitations will be something that will eventually challenge very large structural assembly on orbit. This article addresses the creation of in space assembly (ISA) precepts using a hybrid approach by looking at each phase of the mission or function, i.e., manufacturing, stowage and transport, ISA itself and servicing. Weighted decision matrixes explore the value proposition of advantages and disadvantages with changing architectures.

Dexterity and working with assembly that requires high compliance is the topic of “A Hybrid Soft Material Robotic End-Affector for Reversible In-Space Assembly of Strut Components” from Hammond et al. A soft material robot end-effector, capable of suppling the manipulability, pressure, and temperature requirements for bonding/de-bonding of conical structural components is presented. The authors investigate the feasibility of a hybrid soft robotic end-effector actuated by a unique method (Twisted and Coiled Artificial Muscles or TCAMs) for in-space assembly tasks that require a higher degree of soft manipulability to affect unique assembly techniques.

The idea that a robotic arm or manipulator must be fixed is also challenged from Nair et al. in “Design Engineering a Walking Robotic Manipulator for In-Space Assembly Missions”. In the forthcoming decades, newer infrastructures in the Earth’s orbits, which are much more advanced than the International Space Station (ISS), are needed for *in situ* manufacturing, servicing, and astronomical and observational stations. The authors introduce an innovative dexterous walking robotic system for in-orbit assembly missions which looks at telescope system with an aperture of 25 m as the use case.

The contributions in this Research Topic offer a broad perspective on the multi-dimensional approaches to both execution and architecture for in space assembly. Various strategies and approaches are showcased that address the challenges in execution. These findings encourage further research in this direction and suggest additional investigations orthogonal to the activities to-date.

